# Perioperative Ventilatory Management in Cardiac Surgery: A French Nationwide Survey

**DOI:** 10.1097/MD.0000000000002655

**Published:** 2016-03-07

**Authors:** Marc-Olivier Fischer, Benoît Courteille, Pierre-Grégoire Guinot, Hervé Dupont, Jean-Louis Gérard, Jean-Luc Hanouz, Emmanuel Lorne, the collaboration of ARCOTHOVA, CARGO Groups

**Affiliations:** From the Anesthesiology and Critical Care Department (M-OF, BC, J-LG, J-LH), University Hospital of Caen; EA 4650 (MOF, JLH), Caen Basse-Normandie University, Esplanade de la Paix, Caen; Anesthesiology and Critical Care Department (P-GG, HD, EL), Amiens University Medical Center, Amiens; and INSERM U 1088 (P-GG, HD, EL), University of Picardie Jules Verne, Centre Universitaire de Recherche en Santé, Amiens cedex, France.

## Abstract

Protective ventilation is associated with a lower incidence of pulmonary complications. However, there are few published data on routine pulmonary management in adult cardiac surgery. The present study's primary objective was to survey pulmonary management in this high-risk population, as practiced by anesthesiologists in France.

All 460 registered France-based cardiac anesthesiologists were invited (by e-mail) to participate in an online survey in January–February 2015. The survey's questionnaire was designed to assess current practice in pre-, per-, and postoperative pulmonary management.

In all, 198 anesthesiologists (43% of those invited) participated in the survey. Other than during the cardiopulmonary bypass (CPB) per se, 179 anesthesiologists (91% of respondees) [95% confidence interval (CI): 87–95] used a low-tidal-volume approach (6–8 mL/kg), whereas techniques based on positive end-expiratory pressure and recruitment maneuvers vary greatly from 1 anesthesiologist to another. During CPB, 104 (53%) [95% CI: 46–60] anesthesiologists withdrew mechanical ventilation (with disconnection, in some cases) and 97 (49%) [95% CI: 42–56] did not prescribe positive end-expiratory pressure. One hundred sixty-five (83%) [95% CI: 78–88] anesthesiologists stated that a written protocol for peroperative pulmonary management was not available. Twenty (10%) [95% CI: 6–14] and 11 (5%) [95% CI: 2–8] anesthesiologists stated that they did use protocols for ventilator use and recruitment maneuvers, respectively. The preoperative period (pulmonary examinations and prescription of additional assessments) and the postoperative period (extubation, and noninvasive ventilation) periods vary greatly from 1 anesthesiologist to another.

The great majority of French cardiac anesthesiologists use a low tidal volume during cardiac surgery (other than during CPB per se). However, pulmonary management procedures varied markedly from 1 anesthesiologist to another. There is a clear need for large clinical studies designed to identify best practice in pulmonary management.

## Abstract 2

Supplemental Digital Content is available in the text

## INTRODUCTION

Pulmonary complications occur frequently after cardiac surgery with cardiopulmonary bypass (CPB), and are associated with prolonged hospitalization and additional costs. The pathophysiology of these complications (such as ischemia-reperfusion syndrome, acute lung injury, and/or atelectasis) is complex and multifactorial.^1–3^ Protective mechanical ventilation was first developed for acute respiratory distress syndrome,^4^ and was then successfully extended to abdominal surgery.^5^ Protective ventilation using low tidal volumes is of benefit in cardiac surgery; a number of studies have reported that application of this technique is associated with a lower incidence of pulmonary complications and organ failure and a shorter length of stay in the intensive care unit (ICU).^6,7^ However, studies on ventilatory strategies during CPB^3^ or the postoperative period^8,9^ are controversial, and no clinical guidelines are available on pulmonary management in adult cardiac surgery.

The primary objective of the present French nationwide survey of anesthesiologists was to describe routine pulmonary management in adult cardiac surgery. The starting hypothesis was that the majority of French anesthesiologists use mechanical ventilation with a low tidal volume. The study's secondary objectives were to describe the use of other protective ventilation techniques (positive end-expiratory pressure (PEEP) and lung recruitment maneuvers), pre-and postoperative pulmonary management, and the availability of institutional protocols.

## METHODS

### Ethics

The study's objectives and procedures were approved by the local investigational review board (Comité de Protection des Personnes Nord Ouest III, Caen, France; Chairman Charlotte Gourio) on December 11, 2014 (reference: A14-D67-VOL.23). All 460 registered France-based cardiac anesthesiologists were invited (by e-mails) to participate in an online survey in January–February 2015. The survey's questionnaire was designed to assess current practice in pre-, per-, and postoperative pulmonary management. In view of the anonymous nature of the study, the need for written, informed consent authorization was waived. There were no exclusion criteria. The study complied with the survey-reporting list.^10^

### Study Population

By cross-checking the membership lists of the 2 main French societies for cardiac anesthesiologists (the ARCOTHOVA, Association d’Anesthésistes Réanimateurs du Cœur, du Thorax, et des Vaisseaux and the CARGO, the Cercle des Anesthésistes réanimateurs du Grand Ouest en chirurgie cardiaque) and the AXIS register (Axis-Editions, Saint-Max, France), we e-mailed 460 French anesthesiologists practicing in the field of adult cardiac surgery (at 53 different medical centers) and invited them to participate in the survey. To maximize the response rate, the invitation was sent out 3 times (between January 13, 2015, and February 19, 2015). The e-mail contained a cover letter containing the lead investigator's name and contact details, and information on the sponsoring organization, the study's objectives and the sampling and including the contact name and e-mail of the researcher, the aims of the study and the methodology. The online questionnaire was published using SurveyMonkey (SurveyMonkey, Palo Alto, CA).

### Questionnaire Layout

The questionnaire consisted of 25 multiple-choice questions on perioperative ventilatory management in cardiac surgery. The questions were grouped according to 3 distinct surgical periods: the preoperative period, the peroperative period (with a distinction drawn between CPB per se and the period before and after CBP), and the postoperative period. The questionnaire had been previously tested on a pilot sample of 5 expert cardiac anesthesiologists, to check whether the questions were well understood and to measure the completion time (estimated at between 2 and 3 minutes). The questionnaire is provided in the Supplemental Digital Content (S1;). The participating cardiac anesthesiologists answered the online questionnaire directly and anonymously.

### Endpoints

The primary endpoint was the proportion of cardiac anesthesiologists who performed mechanical ventilation using low tidal volumes (with “low” defined as between 6 and 8 mL/kg, for the purposes of the present survey). We tested the hypothesis whereby the majority (>75%) of French cardiac anesthesiologists used mechanical ventilation with low tidal volumes.

The secondary endpoints were to describe the use of other protective ventilation techniques (defined as PEEP and lung recruitment maneuvers), pre- and postoperative procedures for pulmonary management, and the availability of institutional protocols.

### Statistical Analysis

In view of the relative small number of cardiac anesthesiologists in France, we decided to attempt to survey all of them; hence, a sample size calculation was unnecessary. After all completed questionnaires had been received, they were analyzed using MedCalc software (version 12.5.0, MedCalc Software bvba, Ostend, Belgium). All descriptive data are presented as the number (percentage of survey respondees) [95% confidence interval].

## RESULTS

Of the 460 anesthesiologists contacted, 35 (8%) e-mail addresses were no longer valid. In all, 198 (43%) anesthesiologists participated in the survey [154 (78%) after the first e-mail, 23 (11%) after the second e-mail, and 21 (11%) after the third e-mail]. Among the answered questionnaires, 95.6% [95% CI: 92.7–98.5] of the questions have been fully completed. The survey population's professional and demographic characteristics are summarized in Table 1. Most of the anesthesiologists worked in university medical centers, although the individual levels of experience (years of practice) and levels of activity (number of cardiac operation per year per center) varied greatly.

**TABLE 1:**
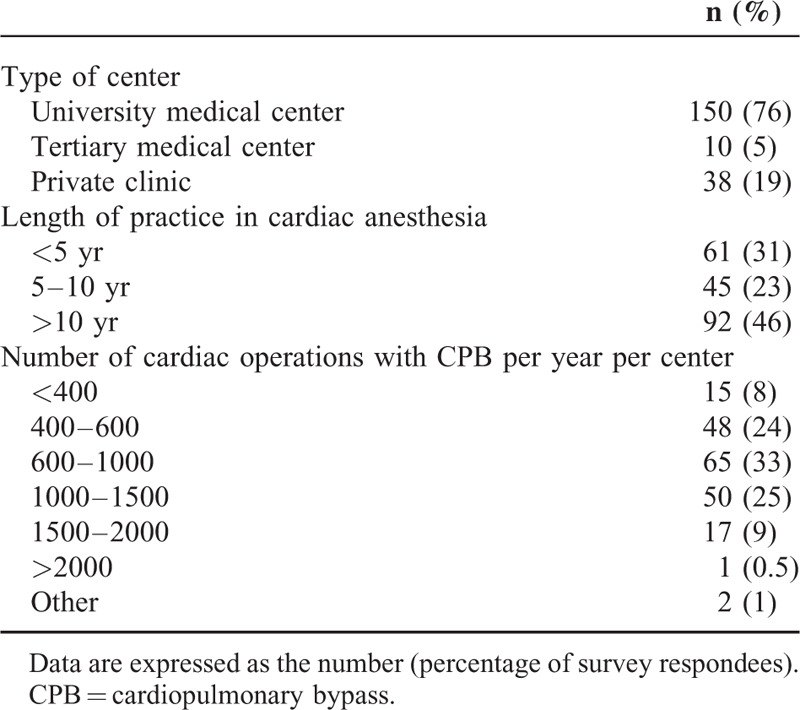
Demographic Data on the Surveyed Anesthesiologists (Respondees, n = 198)

### Preoperative Period

**FIGURE 1:**
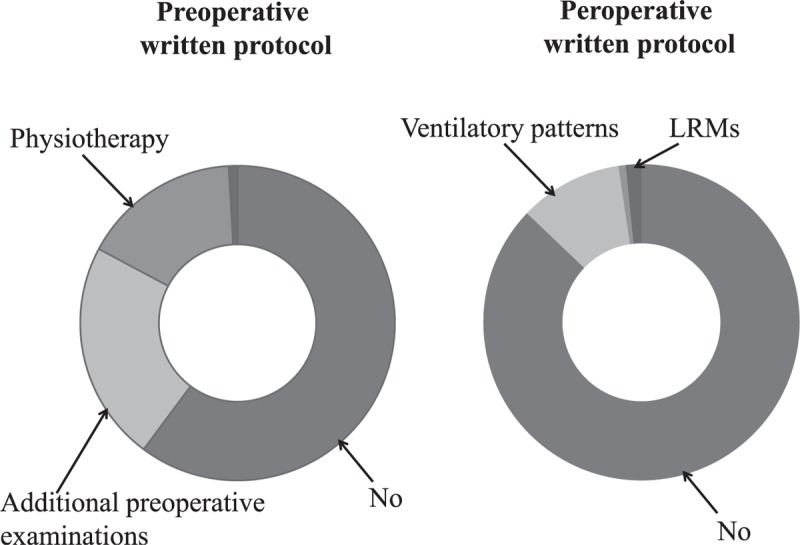
Availability of a written protocol for preoperative pulmonary management (left panel) and peroperative pulmonary management (right panel) in cardiac surgery (n = 194 and 186 respondees, respectively). LRMs = lung recruitment maneuvers.

Data on the pulmonary management techniques used in the preoperative period are summarized in Table 2. Pulmonary examinations were prescribed in the great majority of cases as chest radiography was prescribed in 156 (79%) [95% CI: 73–84] of cases. A preoperative written protocol was not available, available for check, or for physiotherapy according to 133 (68%) [95% CI: 62–74], 50 (25%) [95% CI: 19–31], and 36 (18%) [95% CI: 13–23] anesthesiologists, respectively (Figure 1).

**TABLE 2:**
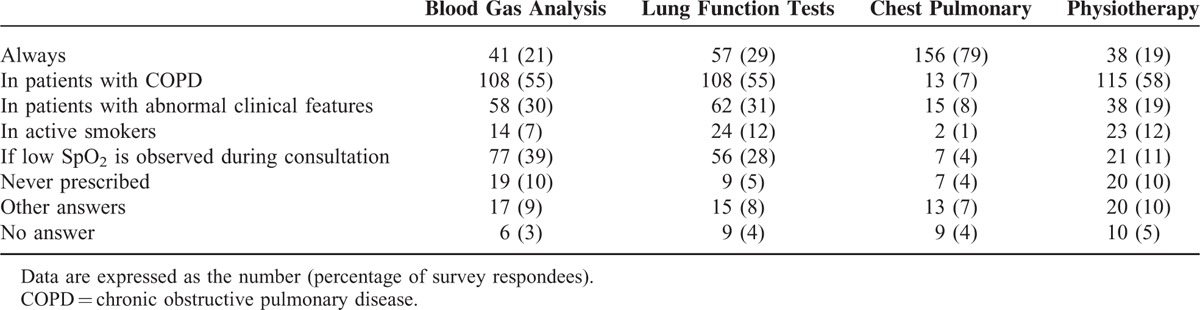
Examinations Prescribed Prior to Cardiac Surgery (n = 198)

### Peroperative Period

**FIGURE 2:**
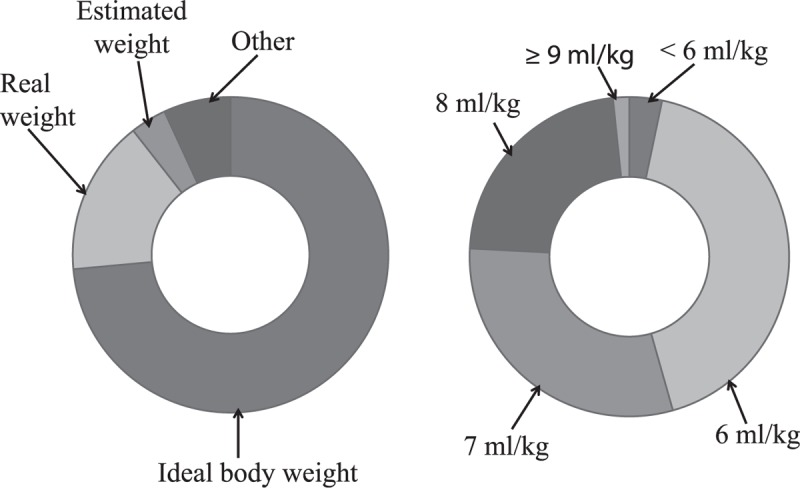
Ventilatory patterns during cardiac surgery (other than during CPB). The figure shows the calculated weight (left panel) and the tidal volume (right panel) (n = 189). CPB = cardiopulmonary by-pass.

**FIGURE 3:**
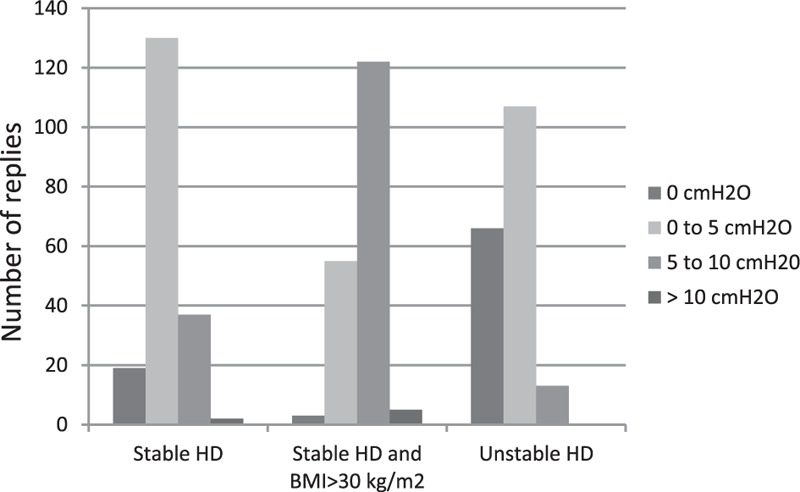
The positive end-expiratory pressure selected for 3 conditions: stable hemodynamic conditions (left panel), stable hemodynamic conditions and BMI >30 kg/m^2^ (middle panel), and unstable hemodynamic conditions (right panel) (n = 189). BMI = body mass index.

**FIGURE 4:**
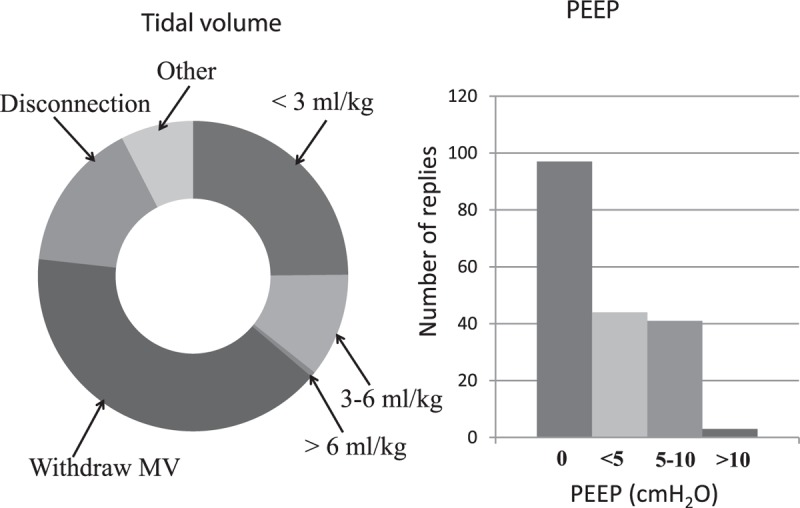
Ventilatory patterns during CPB, showing the tidal volume (left panel) and the postend expiratory pressure (right panel) (n = 186). MV = mechanical ventilation; PEEP = positive end-expiratory pressure.

As mentioned above, 179 (91%) [95% CI: 87–95] anesthesiologists used a low tidal volume. The survey data on selected tidal volume and PEEP prior to CPB are summarized in Figures 2 and 3, respectively. The ventilator patterns used during CPB are shown in Figure 4. One hundred four (53%) [95% CI: 46–60] anesthesiologists stated that they withdrew mechanical ventilation (with disconnection, in some cases) and 97 (49%) [95% CI: 42–56] did not apply PEEP during CPB. The survey data on the use of lung recruitment maneuvers during cardiac surgery and during CPB are summarized in Tables 3 and 4, respectively. Sixty-one (33%) and 8 (4%) anesthesiologists realized lung recruitment maneuvers systematically for all patients during surgery and CPB, respectively. One hundred sixty-five (83%) [95% CI: 78–88] anesthesiologists stated that a written protocol for peroperative pulmonary management was not available. Twenty (10%) [95% CI: 6–14] and 11 (5%) [95% CI: 2–8] anesthesiologists stated that they did not use protocols for ventilator use and lung recruitment maneuvers, respectively (Figure 1).

**TABLE 3:**
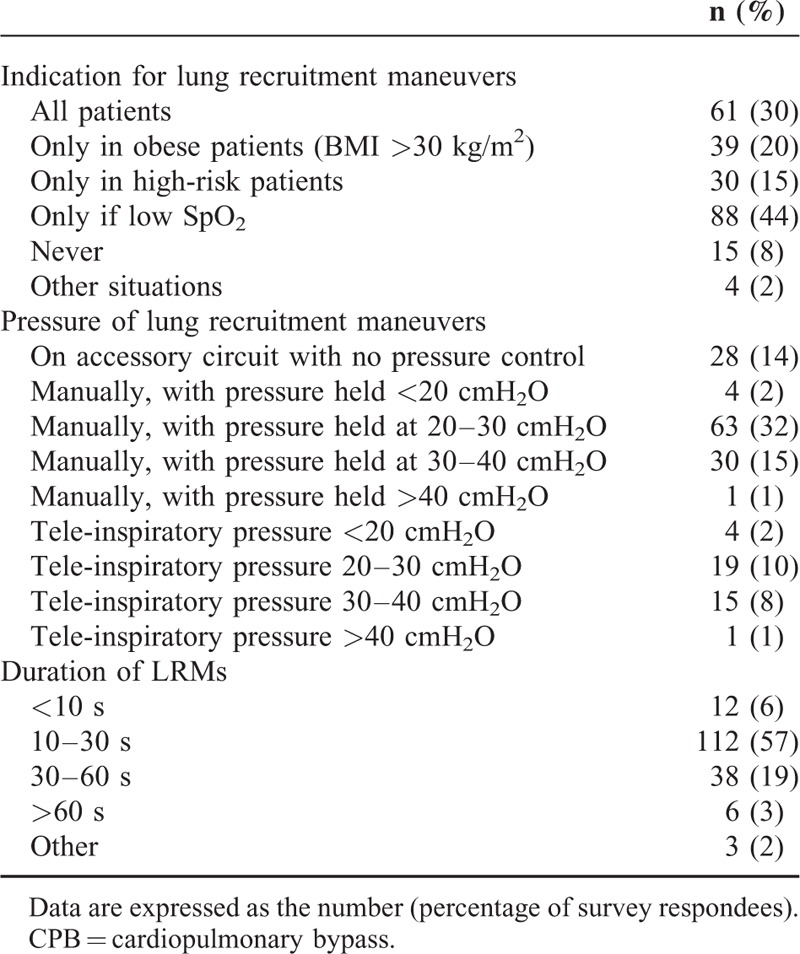
Lung Recruitment Maneuvers During Cardiac Surgery (Except CPB) (n = 184)

**TABLE 4:**
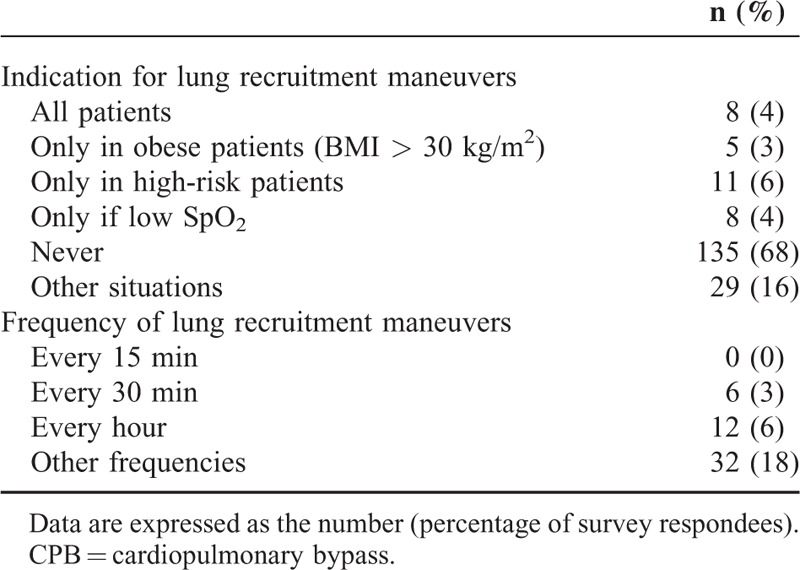
Lung Recruitment Maneuvers During CPB (n = 186)

### Postoperative Period

Extubation was more frequent in ICUs than in the operating theatre. Patients with extubation usually underwent subsequent physiotherapy. In contrast, endotracheal aspiration and noninvasive ventilation were more controversial (Table 5). No relationship was observed according to the endotracheal aspiration practice, and the postoperative noninvasive ventilation strategy.

**TABLE 5:**
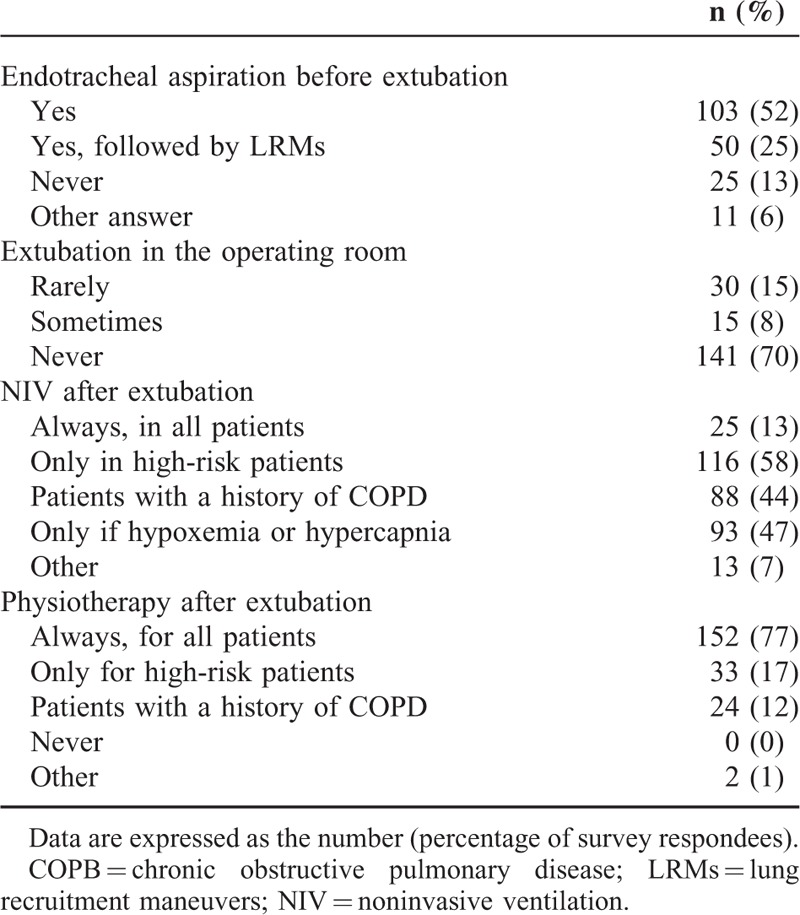
Postoperative Pulmonary Management (n = 186)

The study data are presented in detail in the Supplemental Digital Content (S2;).

## DISCUSSION

The main findings of the present nationwide survey of ventilatory management in cardiac surgery are as follows: the concept of “protective ventilation” using low tidal volumes (based on the ideal body weight) is applied by a large proportion of French anesthesiologists, although PEEP and lung recruitment maneuvers were rarely used; practices in the pre-, per-, and postoperative periods vary markedly from 1 anesthesiologist to another and from 1 center to another; and a written, institutional protocol for pulmonary management in cardiac surgery is rarely available.

We used an online questionnaire to evaluate practice among France-based cardiac anesthesiologists. The present study adopted the 17-item reporting list for quality recently described by Story et al.^10^ Indeed, as has already been described for randomized trials,^11^ systematic reviews,^12^ and observational trials,^13^ a high-quality methodology is essential for surveys of practice in anesthesia^10^ and in critical care.^14^ The latter publications emphasized the inconsistent nature of survey reporting in the literature, which compromises the clarity and reproducibility of survey reports. Our compliance with these guidelines strengthened the survey's internal validity. Moreover, the great majority of respondents gave full answers to all questions.

Our present results showed that a great majority of anesthesiologists used a low tidal volume (between 6 and 8 mL/kg) during cardiac surgery (other than during CPB per se). Although no guidelines on pulmonary management in cardiac surgery are available, 2 recent studies emphasized the clinical benefit of low-tidal-volume ventilation in this population; this may explain the high level of uptake by the surveyed anesthesiologists.^6,7^ In a randomized controlled trial, a low-tidal-volume strategy (6 mL/kg, vs. a high volume of 10 mL/kg) resulted in a lower incidence of mechanical ventilation at 6 hours after intubation, and a lower reintubation rate after surgery.^6^ In a large, observational study, a tidal volume above 10 mL/kg of predicted body weight was found to be a significant risk factor for organ failure, and prolonged the length of stay in the ICU (particularly for women and obese patients).^7^ Indeed, high tidal volumes have been associated with an increase in local and systemic inflammation, and a decrease in mean arterial pressure compared with low tidal volume ventilation.^7^ Last, the results of the recent IMPROVE study further emphasized the value of a lung-protective ventilation strategy by evidencing better clinical outcomes and reduced health care utilization.^5^ In contrast, ventilation strategies were very heterogeneous during CPB-probably because of the lack of data during this period,^3^ and the constraints induced by collaboration between anesthesiologists and surgeons.^15^ Moreover, our survey results indicate that other components of a protective ventilation strategy (such as the lung recruitment maneuvers and the PEEP, concerning their adjustment and their use) vary greatly from 1 anesthesiologist to another and from 1 center to another. This heterogeneity may be due to the absence of clinical trial data on lung recruitment maneuvers in cardiac surgery,^16^ the contrasting reports on the impact of PEEP on pulmonary function,^3^ and the putative interactions between PEEP and both right ventricular function^17^ and diastolic left ventricular function.^18^ However, the proportion of anesthesiologists using both lung recruitment maneuvers and PEEP, especially during CPB, might be low in the present survey, but their beneficial effects and their modalities have not been reported for cardiac surgical patients. One recent work conducted in ARDS critically ill patients suggested that lung recruitment maneuvers duration of 10 seconds (pressure 40 cmH_2_O) could be sufficient.^19^ For patients without preexisting lung injury in abdominal surgery, the IMPROVE study using lung recruitment maneuvers (30 cmH_2_O during 30 seconds, every 30–45 minutes), PEEP (6–8 cmH_2_O), and low tidal volumes (6–8 mL/kg) showed a decrease in postoperative lung morbidity and hospital length of stay.^5^

Our present results showed that practice during the preoperative period varies greatly from 1 anesthesiologist to another. The absence of an institutional protocol might explain these results (at least in part); guidelines would help anesthesiologists to adopt a coherent approach (especially with regard to preoperative assessments). Similar guidelines are available for noncardiac surgery,^20,21^ and so national datasets might improve the clarity and uniformity of patient management and help to avoid costly reassessments.^20^

For the postoperative period, our survey results prompted similar findings. A recent study demonstrated that a standardized protocol increased the likelihood of early extubation and was not associated with elevated reintubation rates.^22^ Fast-track cardiac care for adult cardiac surgery patients appeared to be safe, and decreased the time to extubation and the length of stay in the ICU.^23,24^

The present study had several limitations. First, the survey was declarative, and 198 (43%) of the invited anesthesiologists participated in the study. Among the answered questionnaires, 95.6% [95% CI: 92.7–98.5] of the questions have been fully completed, and could be responsible of a minima interpretation bias. Although our questionnaire has been tested in pilot experiment and contained only multiple-choice questions (to speed completion of the survey), the response rate was below the value of 60% recommended by the survey reporting guidelines.^10^ However, our participation was higher than the value of around 20% reported for a postal survey.^25^ The potential for differences between respondents and nonrespondents (e.g., the absence of protective ventilation or the use of high tidal volumes) cannot be ruled out and may have led to overestimation of the use of low-tidal-volume ventilation in the present survey with a risk of interpretation bias. Second, it is known that the order of questions can influence the answers to the subsequent questions (referred to as an “anchoring effect”).^26,27^ Ideally, the questions should be presented in random order; however, we considered that the survey's coherence would be affected by this approach. Last, the survey did not seek to assess the impact of the various practices described in the present study. To date, no relationship has been found between pulmonary management in cardiac surgery and the patient outcome.

In conclusion, this French nationwide survey showed that the great majority of anesthesiologists use low tidal volumes (between 6 and 8 mL/kg) during cardiac surgery (other than during CPB per se). However, other pulmonary management practices vary markedly from 1 anesthesiologist to another and from 1 center to another. There is a clear need for large-scale trials designed to define the optimal ventilatory strategy and to inform the preparation of clinical guidelines.

## Supplementary Material

Supplemental Digital Content

## Supplementary Material

Supplemental Digital Content
