# Supplementary material for: Perioperative Ventilatory Management in Cardiac Surgery: A French Nationwide Survey
Source: Medicine (Baltimore). 2016 Mar 7;95(9):e2655. doi: 10.1097/MD.0000000000002655 (PMC4782837; doi:10.1097/MD.0000000000002655)
Supplement: Supplemental Digital Content [file medi-95-e2655-s001.docx]

**Supplemental data 1: Questionnaire**

**This is a French National Survey about the ventilation practices for adult cardiac surgery. The ARCOTHOVA and the CARGO groups supported this work.**

**This survey aims to inform practice regarding ventilation in cardiac surgery in France.**

**This survey is anonymous.**

**Following questions concern the anaesthesia for cardiac surgery in adults.**

**When possible, results will be sending to you as soon as possible, using the mailing list**

**This work could conduct to a publication.**

For any question, you can contact us, using the following Email:

[marcolivierfischer@yahoo.fr](mailto:marcolivierfischer@yahoo.fr)

Dr Marc-Olivier Fischer, Anesthesiologist, University Hospital of Caen, France

**GENERAL INFORMATIONS**

**1. What is your employment status?**

[] Internal

[] Clinical Manager / Assistant anesthesiologist

[] Qualified anesthesiologist

[] Assistant of Professor/Professor

[] Other

**2. What is the type of your medical center?**

[] Public

[] Private clinic

[] Non-Profit Private

**3. How long is your experience in cardiac anesthesiology ?**

[] <5 years

[] 5-10 years

[]> 10 years

**4. How many cardiac surgery with cardiopulmonary bypass perform your team each year?**

[] <400

[] 400-600

[] 600-1000

[] 1000-1500

[] 1500-2000

[]> 2000

**5. Is there in your medical center an available protocol in respiratory management for cardiac anesthesiology before surgery?**

[] No

[] Yes, for prescribing the preoperative exams

[] Yes, for prescribing the preoperative physiotherapy

[] Other

**PRE-OPERATIVE**

**6. In the PRE-OPERATIVE period, do you prescribe always blood gas analysis? If not, in what case (s) do you prescribe one?**

[] Yes

[] No. Only if acute and chronic pulmonary disease

[] No. Only if abnormal clinical features

[] No. Only if active smokers

[] No. Only if low SpO_2_ during anaesthesia consultation

[] No. Never prescribed

**7. In the PRE-OPERATIVE period, do you prescribe systematically lung function tests? If not, in what case (s) do you prescribe one?**

[] Yes

[] No. Only if acute and chronic pulmonary disease

[] No. Only if abnormal clinical features

[] No. Only if active smokers

[] No. Only if low SpO_2_ during anesthesia consultation

[] No. Never prescribed

**8. In the PRE-OPERATIVE period, do you prescribe systematically chest radiography? If not, in what case (s) do you prescribe one?**

[] Yes

[] No. Only if acute and chronic pulmonary disease

[] No. Only if abnormal clinical features

[] No. Only if active smokers

[] No. Only if low SpO_2_ during anaesthesia consultation

[] No. Never prescribed

**9. In the PRE-OPERATIVE period, do you prescribe systematically respiratory physiotherapy? If not, in what case (s) do you prescribe one?**

[] Yes

[] No. Only if acute and chronic pulmonary disease

[] No. Only if abnormal clinical features

[] No. Only if active smokers

[] No. Only if low SpO_2_ during anaesthesia consultation

[] No. Never prescribed

**PER-OPERATIVE before CEC**

**10. Is there a written protocol for the ventilatory patterns during surgery other than during cardiopulmonary bypass ?**

[] No

[] Yes, for the ventilator patterns (tidal volume and/or the post end-expiratory pressure)?

[] Yes, for the lung recruitment maneuvers ?

[] Other

**11. During surgery, how do you choice the tidal volume on the ventilator?**

**Or (depending on the response 10): what tidal volume goal you set yourself?**

[] <6 ml/kg

[] 6 ml/kg

[] 7 ml/kg

[] 8 ml/kg

[] 9 ml/kg

[] 10 ml/kg

[] > 10 ml/kg

[] Other

**12. What weight do you use?**

[] The theoretical ideal body weight calculated for each patient

[] The patient's real weight

[] The estimated weight of the patient

[] I do not take into account the weight for each patient

[] Other

**13. What positive end-expiratory pressure (PEEP) set up immediately in a patient hemodynamically stable?**

[] 0 cmH_2_O

[] <5 cmH_2_O

[] 5-10 cmH_2_O

[]> 10 cmH_2_O

**In an obese patient (BMI > 30 kg/m^2^) ?**

[] 0 cmH_2_O

[] <5 cmH_2_O

[] 5-10 cmH_2_O

[]> 10 cmH_2_O

**In a patient with unstable hemodynamic?**

[] 0 cmH_2_O

[] <5 cmH_2_O

[] 5-10 cmH_2_O

[]> 10 cmH_2_O

**14. Do you make systematic lung recruitment maneuvers (excluding complications) during this period ?**

[] Yes

[] Never

If so to what (s) situation?

[] For all patients

[] Only in obese patients (BMI > 30 kg/m^2^)

[] Only in high-risk patients of postoperative respiratory complications

[] Only if low SpO_2_

**15. In what ways do you perform these lung recruitment maneuvers?**

[] On accessory circuit with no pressure control

[] Manually, with pressure held < 20 cmH_2_O

[] Manually, with pressure held at 20 to 30 cmH_2_O

[] Manually, with pressure held at 30 to 40 cmH_2_O

[] Manually, with pressure held > 40 cmH_2_O

[] Tele-inspiratory pressure < 20 cmH_2_O

[] Tele-inspiratory pressure 20-30 cmH_2_O

[] Tele-inspiratory pressure 30-40 cmH_2_O

[] Tele-inspiratory pressure >40 cmH_2_O

[] Other

**16. For how long do you do perform the lung recruitment maneuver?**

[] <10 sec

[] 10 to 30 sec

[] 30 to 60 sec

[] > 60 sec

[] Other

**DURING CARDIOPULMONARY BYPASS**

**17. What tidal volume set you on the ventilator during the cardiopulmonary by-pass?**

[] <3 ml/kg

[] 3-6 ml/kg

[] > 6 ml/kg

[] Withdraw mechanical ventilation without disconnection

[] Disconnection of the ventilator

[] Other

**18. Do you carry positive end-expiratory pressure during CPB?**

[] Yes

[] No

**19. If yes, what level of PEEP did you prescribe during CPB?**

[] <5 cmH_2_0

[] 5 to 10 cmH_2_0

[] > 10 cmH_2_0

[] Other

**20. Do you carry lung recruitment maneuvers during CPB?**

[] Yes

[] Never

**If so, in what (s) situation?**

[] For all patients

[] Only in obese patients (BMI > 30 kg/m^2^)

[] Only in high-risk patients of postoperative respiratory complications

[] Only if low SpO_2_

**21. When did you carry lung recruitment maneuvers during CPB?**

[] Every 15 min

[] Every 30 min

[] Every hour

[] Other frequencies

**POST-OPERATIVE**

**22. Did you carry the extubation in operating room?**

[] Never

[] Rarely

[] Sometimes

[] Often

[] Always

**23. Before extubation, do you perform endotracheal aspiration?**

[] Yes

[] Yes followed by lung recruitment maneuvers

[] No, never

**24. In which case (s) do you use non-invasive ventilation after surgery?**

[] Always in all patients

[] In patients at high risk for respiratory complications

[] Patients with a history of COPD

[] Only in cases of hypoxemia and / or hypercapnia

[] Never

[] Other

**25. In which case (s) do you prescribe physiotherpy for respiratory postoperative cardiac surgery?**

[] Always for all patients

[] In patients at high risk for respiratory complications

[] Patients with a history of COPD

[] Never

[] Other
